# Proline isomerization in the C-terminal region of HSP27

**DOI:** 10.1007/s12192-017-0791-z

**Published:** 2017-05-25

**Authors:** T. Reid Alderson, Justin L. P. Benesch, Andrew J. Baldwin

**Affiliations:** 1 Department of Chemistry, Physical and Theoretical Chemistry Laboratory, University of Oxford, South Parks Road, Oxford, OX1 3QZ UK; 2 Laboratory of Chemical Physics, National Institute of Diabetes and Digestive and Kidney Diseases, National Institutes of Health, Bethesda, MD 20892 USA

**Keywords:** Molecular chaperones, Small heat-shock proteins, *cis*-*trans* proline isomerization, Nuclear magnetic resonance spectroscopy, Intrinsically disordered proteins

## Abstract

In mammals, small heat-shock proteins (sHSPs) typically assemble into interconverting, polydisperse oligomers. The dynamic exchange of sHSP oligomers is regulated, at least in part, by molecular interactions between the α-crystallin domain and the C-terminal region (CTR). Here we report solution-state nuclear magnetic resonance (NMR) spectroscopy investigations of the conformation and dynamics of the disordered and flexible CTR of human HSP27, a systemically expressed sHSP. We observed multiple NMR signals for residues in the vicinity of proline 194, and we determined that, while all observed forms are highly disordered, the extra resonances arise from *cis*-*trans* peptidyl-prolyl isomerization about the G193-P194 peptide bond. The *cis*-P194 state is populated to near 15% at physiological temperatures, and, although both *cis*- and *trans*-P194 forms of the CTR are flexible and dynamic, both states show a residual but differing tendency to adopt β-strand conformations. In NMR spectra of an isolated CTR peptide, we observed similar evidence for isomerization involving proline 182, found within the IPI/V motif. Collectively, these data indicate a potential role for *cis*-*trans* proline isomerization in regulating the oligomerization of sHSPs.

**Electronic supplementary material:**

The online version of this article (doi:10.1007/s12192-017-0791-z) contains supplementary material, which is available to authorized users.

## Introduction

**Fig. 1:**
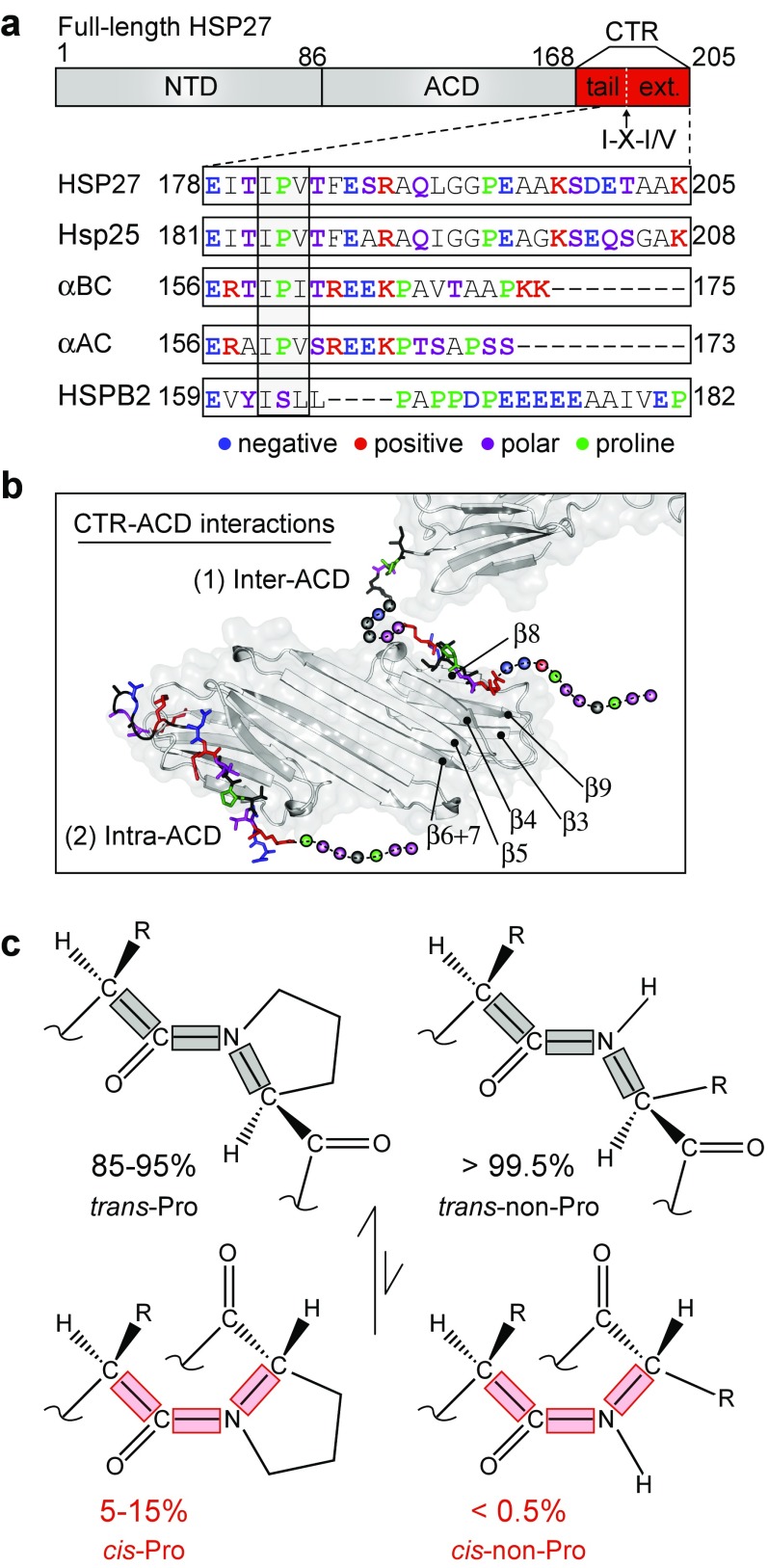
The disordered C-terminal region of HSP27 contains an abundance of charged and proline residues. **a** HSP27 comprises three regions: an N-terminal domain (*NTD*), an α-crystallin domain (*ACD*), and a C-terminal region (*CTR*). The CTR is further broken down into the C-terminal tail, which includes residues up to the highly conserved IXI/V motif, and the C-terminal extension (ext.) from end of the IXI/V motif through the C-terminus. The *inset* depicts an amino acid sequence alignment of CTRs from various mammalian sHSPs. Shown here are human HSP27, murine Hsp25, human αB-crystallin (αBC), human αA-crystallin (αAC), and human HSPB2. Negatively charged residues are *colored blue*, positive residues are *red*, polar residues are *purple*, and proline residues are *green.* The IXI/V motif is *boxed*. **b** Interactions between the CTR and ACD involve both intra- and inter-ACD binding. Inter-ACD interactions are observed in a crystal structure of αA-crystallin (PDB ID: 3L1G), with the C-terminal residues colored as above. Intra-ACD interactions are found in a second αA-crystallin structure (PDB: 3N3E), but the C-terminal residues are shown on 3L1G for clarity. Amino acids from the CTR that were not observed in the crystal structure are shown as *spheres*. **c** Schematic depicting the propensity for X-Pro peptide bonds to form either *trans*- (*black*) or *cis*-proline (*red*) conformations. On the right is shown an X-X peptide bond (i.e., non-Pro) for comparison

Small heat-shock proteins (sHSPs) exist in all kingdoms of life and exhibit a diverse range of cellular roles from preventing protein aggregation, to upholding cytoskeletal integrity, and regulating apoptosis (Kampinga et al. 2015). Despite relatively small molecular masses, sHSPs typically assemble into large, dynamic oligomers of varying sizes and degrees of polydispersity (Mchaourab et al. 2009; Hilton et al. 2013b; Haslbeck and Vierling 2015). The mechanisms of oligomerization among the 10 human sHSPs (HSPB1–10) remain poorly understood, but the self-assembly process involves a variety of inter- and intra-molecular contacts between the α-crystallin domain (ACD) and its flanking N-terminal domain (NTD) and C-terminal region (CTR). The CTR is subdivided into the tail and the extension, which respectively include residues up to a well-conserved IXI/V motif, and beyond (Fig. 1a). Crystal structures of non-mammalian sHSPs revealed that the IXI/V motif docks into a hydrophobic groove in the ACD of a neighboring subunit (Mchaourab et al. 2009; Hilton et al. 2013b; Haslbeck and Vierling 2015). Crystal structures of truncated constructs suggest that similar contacts are made in oligomeric mammalian sHSPs, and that docking of the CTR can in principle occur bi-directionally (Laganowsky et al. 2010) and intra- or inter-monomer (Laganowsky and Eisenberg 2010) (Fig. 1b). However, it is evident that in solution these interactions are transient (Baldwin et al. 2012), with the CTRs overall being predominately disordered in solution (Carver and Lindner 1998; Delbecq and Klevit 2013; Hochberg and Benesch 2014; Treweek et al. 2015).

Evidence for this flexibility of the CTR has come primarily from solution-state nuclear magnetic resonance (NMR) spectroscopy, which is well suited to studying unstructured regions within proteins. Disorder in the CTR has been observed in αA- and αB-crystallin (human HSPB4 and HSPB5, respectively) (Carver et al. 1992), HSP27 (HSPB1) (Carver and Lindner 1998), murine Hsp20 (van de Klundert et al. 1998), murine Hsp25 (Carver et al. 1995a), and *Saccharomyces cerevisiae* Hsp26 (Benesch et al. 2010). Detailed interrogation of the CTR in αB-crystallin has revealed that, in solution above 0 ºC, the IPI/V motif is transiently bound to the ACD at only a few percent of the total (Baldwin et al. 2011a; Baldwin and Kay 2012), and that the independent binding of two CTRs, either in an intra- or inter-molecular manner, triggers the dissociation of a monomer from an oligomer (Baldwin et al. 2011b; 2012). The strength and kinetics of the interaction are responsive to amino acid substitutions (Smulders et al. 1996; Hilton et al. 2013a), such that it confers some selectivity between sHSPs (Delbecq et al. 2012). These interactions appear to be finely tuned to regulate both sHSP assembly and hetero-oligomerization (Hochberg and Benesch 2014; Delbecq et al. 2015).

HSP27 is a systemically expressed sHSP that plays a central role in maintaining protein homeostasis (Arrigo 2001), but its elevated expression often results in poor prognosis in multiple cancers (Ciocca and Calderwood 2005). Numerous post-translational modifications (PTMs) have been reported in the CTR of HSP27 (Blakytny et al. 1997; Sakamoto et al. 2002; Nagaraj et al. 2012; Ylikallio et al. 2015), some of which directly influence biological activity. Notably, argipyrimidation of R188 in the CTR of HSP27 prevents caspase-3- and caspase-9-mediated apoptosis, prolonging the life of human carcinoma cells (Sakamoto et al. 2002). Moreover, mutations in the CTR of HSP27 are implicated in neuromuscular and neurodegenerative diseases including amyotrophic lateral sclerosis (Capponi et al. 2016) and Charcot-Marie-Tooth disease (Evgrafov et al. 2004; Kijima et al. 2005; D’Ydewalle et al. 2011; Chalova et al. 2014; Ylikallio et al. 2015). The high density of PTMs and disease-associated mutations in the CTR of HSP27 allude to a significant functional role for this region in health, and malfunction in disease.

Structural information about HSP27 remains relatively limited, as it assembles into a polydisperse ensemble of oligomers centered around ~600 kDa (Jovcevski et al. 2015). However, it comprises an ~80-residue ACD that folds into an immunoglobulin-like domain (Baranova et al. 2011; Hochberg et al. 2014; Rajagopal et al. 2015) (Fig. 1b). The CTR contains an abundance of charged, polar, and proline residues (Fig. 1a), which are known to promote disordered backbone conformations (Theillet et al. 2014), and has been crystallized bound to the ACD (Hochberg et al. 2014) (Fig. 1b). In crystal structures of αA-, αB-crystallin, and HSP27 ACD-CTR complexes, the CTR forms an extended conformation in which the I-P peptide bond in the IPI/V motif adopts the *trans*-conformation, enabling penetration of the adjacent residues into the hydrophobic groove of the ACD. Here, we have probed the conformation and dynamics of the HSP27 CTR by means of solution-state NMR spectroscopy. We report that the CTR is predominately disordered, and adopts distinct unfolded structural ensembles that interconvert on the seconds timescale or longer via *cis-trans* peptidyl-prolyl isomerization at its two proline residues. Notably, we show that the *cis*-conformers are populated 20- to 30-fold higher than expected for a fully disordered peptide of this sequence without any proline residues. Given the importance of the CTRs of mammalian sHSPs in assembly and recognition (Delbecq and Klevit 2013; Hochberg and Benesch 2014; Treweek et al. 2015), we speculate that proline isomerization may play a role in regulating the assembly of sHSPs.

## Results

### The C-terminal region of HSP27 populates multiple conformations

**Fig. 2:**
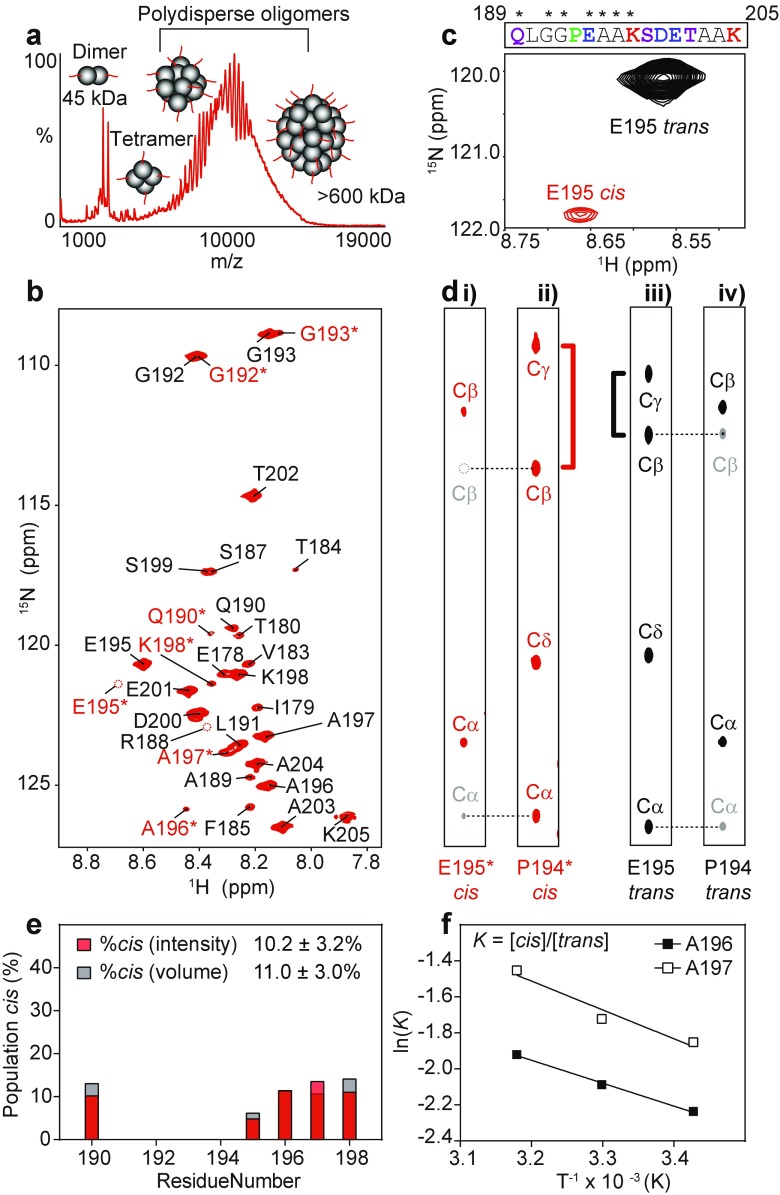
*Cis-trans* proline isomerization about the G193-P194 peptide bond in HSP27. **a** Native mass spectrum of 25 μM HSP27 in 200 mM ammonium acetate, pH 6.9. The *y*-axis depicts signal intensity and the *x*-axis displays the mass-to-charge ratio. **b** 2D ^1^H-^15^N HSQC spectrum of 2 mM [*U*-^13^C,^15^N]-HSP27 at 298 K in 30 mM NaH_2_PO_4_, 100 mM NaCl, 2 mM EDTA, 2 mM NaN_3_, pH 7. Each peak corresponds to an N–H bond and resonance assignments are listed next to each peak. Residues that are colored in *red* and labeled with an *asterisk* (*) indicate doubled peaks. The *dashed circles* indicate resonances that are weak and not observable at the plotted contour level. See Fig. S1 for a lower contour HSQC spectrum. **c** Residues that exhibit two peaks are shown with *asterisks* (*) above their one-letter amino acid code. A zoomed-in region from **b**, which shows the major (*black*) and minor (*red*) peaks that arise from E195. **d** 2D ^1^H-^13^C strip plots taken from 3D HNCACB (*i.* and *iv.*) and C(CO)NH (*ii.* and *iii.*) spectra at the ^15^N frequency corresponding to E195 or E195*. In the HNCACB spectrum, the ^13^Cα and ^13^Cβ nuclei from E195 (*red/black*) and P194 (*gray*) are indicated with *dashed lines* to reveal their corresponding positions in the C(CO)NH spectrum taken at the E195 ^15^N frequency. The difference between ^13^Cβ and ^13^Cγ chemical shifts in proline residues is diagnostic of *cis*- or *trans*-proline bonds. In the minor state, the ^13^Cβ and ^13^Cγ chemical shift difference is ~10 ppm (*red*), indicative of a *cis*-conformation, whereas in the major state this difference is only ~5 ppm (*black*), indicative of a *trans*-conformation. **e** The population of *cis*-P194 at 298 K is shown for each residue with well-resolved *cis* and *trans* peaks in the ^1^H-^15^N HSQC spectrum. Both the peak intensity (*red*) and integrated volume (*gray*) are shown, and the average value ±1 standard deviation is listed. **f** The natural logarithm of the equilibrium constant for *cis*-*trans* isomerization about the G193-P194 peptide bond is shown as a function of temperature. This analysis yields a change in enthalpy (ΔH) and change in entropy (ΔS) of 13.5 kJ mol^−1^ and 3.5 J mol^−1^ K^−1^, respectively, upon formation of the *cis*-G193-P194 peptide bond

To analyze the CTR of HSP27 by solution-state NMR spectroscopy, we recombinantly expressed and purified human HSP27 uniformly labeled ([*U*-]) with ^13^C and ^15^N, and verified that it formed polydisperse oligomers. The native mass spectrum of this sample revealed many overlapping signals that arise from the heterogeneous distribution of oligomeric states (Fig. 2a). Due to significant overlap, the individual signals in the 6000–15,000 *m*/*z* region cannot be assigned to particular oligomeric states, yet arise from large oligomers that range between ~100 and 700 kDa, consistent with previous measurements on unlabeled protein (Aquilina et al. 2013; Jovcevski et al. 2015).

We next interrogated the conformations and dynamics of the CTR using NMR spectroscopy. Using [*U*-^13^C,^15^N]-HSP27, we recorded a two-dimensional (2D) ^1^H-^15^N heteronuclear single-quantum coherence (HSQC) spectrum, which correlates the chemical shifts of ^1^H nuclei that are covalently attached to ^15^N nuclei (e.g., amide and amine bonds) (Fig. 2a). We observed sharp resonances with limited dispersion in the ^1^H dimension, indicating that these resonances arise from dynamically disordered residues lacking a fixed tertiary structure. In order to assign the observed resonances to specific HSP27 residues, we recorded three-dimensional (3D) NMR spectra from [*U*-^13^C, ^15^N]-labeled HSP27 that correlate ^13^C, ^15^N, and ^1^H^N^ chemical shifts of neighboring amino acids whose nuclei are linked through a network of covalent bonds (Sattler et al. 1999). This allowed us to unambiguously assign the observed resonances to residues E178–K205 (Fig. 2b). These data therefore reveal that the final 28 residues of HSP27, including the IPV motif, are predominately disordered in solution.

### Proline residues in the HSP27 CTR undergo *cis-trans* isomerization

In our assignments, we noticed that a subset of HSP27 CTR residues yielded two unique signals per residue, with appreciably different signal intensities. This reveals two CTR conformational ensembles that interconvert with each other relatively slowly, on a timescale of seconds or longer. Interestingly, the subset of residues with two NMR signals all clustered near a single proline residue, P194 (Fig. 2c). In a disordered chain, the majority of amino acids are expected to form peptide bonds that predominantly adopt the *trans* conformation (>99.5%) (Fig. 1c, right). By contrast, peptide bonds involving proline residues (X-Pro) have an increased population of the *cis* conformation (5-15%) (Weiss et al. 1998; Theillet et al. 2014) (Fig. 1c, left). The inter-conversion of the two forms involves the rearrangement of a covalent bond, which results in a substantially slower rate of exchange, typically on the seconds timescale or slower under physiological conditions (Grathwohl and Wüthrich 1981; Weiss et al. 1998; Wedemeyer et al. 2002). Our observation of multiple resonances for residues in the vicinity of P194 (Fig. 2c) hence alluded to potential *cis*-*trans* proline isomerization about the G193-P194 peptide bond.

We directly assessed the possibility of a *cis*-Pro conformation in the G193-P194 peptide bond by analyzing the ^13^C side-chain chemical shifts in P194 (Fig. 2d). The difference in ^13^C chemical shifts between Pro-Cβ and Pro-Cγ nuclei is highly diagnostic of *cis* (~10 ppm) or *trans* (~5 ppm) peptide bond conformations (Schubert et al. 2002; Shen and Bax 2010). Using a 3D C(CO)NH experiment (Grzesiek et al. 1993), we correlated adjacent side-chain ^13^C chemical shifts to show clear relations between resonances. Such an experiment reveals that the two resonances from E195 can be attributed to being directly adjacent to either the *cis* or *trans* form of P194 (Fig. 2d). This finding is independently supported by a calculation using PROMEGA (Shen and Bax 2010), which predicts *cis*- or *trans*-Pro conformations based on ^13^C chemical shifts. Similarly, additional minor peaks from other residues near P194 arise from the formation of the *cis*-P194 bond (Fig. S1). Together, these data clearly demonstrate that proline isomerization occurs about the G193-P194 peptide bond.

Signal intensity coming from the second proline residue (P182) in the CTR of HSP27 in the 3D C(CO)NH experiment was insufficient to determine any populations of *cis*-P182, likely owing to its proximity to the slowly tumbling oligomer bulk. To address the likelihood of isomerization about the I181-P182 peptide bond, we therefore prepared a non-isotopically enriched peptide comprising residues E178–E186. In the methyl region of the ^1^H-^13^C HSQC spectrum of this peptide, we would expect eight resonances in the absence of isomerization. However, we observed 15 peaks arising from methyl-bearing residues (Fig. S2). The appearance of these additional resonances demonstrates that the peptide adopts multiple structural ensembles, with the most probable cause being proline isomerization. Given that the IPV motif is largely unbound in the oligomers, it is highly likely that P182 exists in both *cis* and *trans* forms.

### The *cis*-P194 state is populated to approximately 15% in HSP27 oligomers

The intensities of NMR signals are related to the number of nuclei resonating at a given chemical shift and therefore reflect the population of a given conformation, provided the two forms undergo similar dynamics. We therefore extracted the peak intensities from both the *cis-* and *trans*-P194 conformations, and calculated the population of the *cis* state to be ~10% at 25 °C (Fig. 2e). In an independently prepared sample, we used two well-resolved and relatively intense resonances from adjacent resonances that we could unambiguously attribute to the *cis* or *trans* forms (A196, A197) to calculate the equilibrium constant *K* = [*cis*]/[*trans*] at 20, 30, and 40 °C in order to probe the enthalpic and entropic contributions to formation of the *cis*-P194 state (Fig. 2f). The population of the *cis* state increased to ~15% at 40 °C, our closest measure to body temperature. This variation in equilibrium position could be explained by the process being reversible and characterized by constant enthalpy and entropy values over the temperature range studied. A Van’t Hoff plot of the resultant equilibrium constant at each temperature indicates that the *cis*-P194 conformation is enthalpically disfavored (~10 kJ mol^−1^), but entropically favored (3 J K^−1^ mol^−1^) over the *trans*-P194 state (Fig. 2f). These results, derived from the full-length protein in heterogeneous oligomeric forms, are consistent with data obtained from small, proline-containing oligopeptides (Grathwohl and Wüthrich 1981; Eberhardt et al. 1993; Troganis et al. 2000).

### Proline isomerization affects the propensity for residual β-strand structure in the CTR

**Fig. 3:**
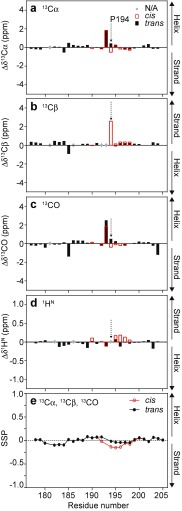
Secondary chemical shifts, Δδ, probe residual structure in the C-terminal region of HSP27. Secondary chemical shifts are defined as the difference between the experimentally measured chemical shift of a given nuclei in a given residue and the same nuclei from the same residue in a random coil conformation, typically extracted from a database of known values. Shown here are the secondary chemical shifts for ^13^Cα (**a**), ^13^Cβ (**b**), ^13^CO (**c**), and ^1^H^N^ (**d**) nuclei in HSP27. The *arrow* depicts the position of P194 in the amino acid sequence. Note that artificially large values are observed for G193 due to its *i*-1 position to P194. **e** The ^13^C chemical shifts from the CTR of HSP27 were used as input to calculate the secondary structure propensity (SSP) of this region. The *y*-axis depicts the propensity to populate α-helical (+1.0) or β-strand (−1.0) conformations, with a value of, e.g., −0.2 corresponding to 20% β-strand. The *cis*-P194 state is shown in *red* and the *trans*-P194 state in *black*

The formation of *cis*-Pro can introduce β-hairpin conformations (Wedemeyer et al. 2002), and so we characterized the effect of the *cis*-P194 bond on backbone conformations in the CTR of HSP27. NMR chemical shifts from backbone and side-chain nuclei are highly sensitive to dihedral angles, hydrogen bonds, and secondary structure. Although both *cis*- and *trans*-P194 CTR ensembles are disordered, we sought to determine if they adopt an entirely random conformation, or if they have a tendency to adopt residual local order. We compared the conformations of the *cis*- and *trans*-P194 states by calculating secondary chemical shifts (Δδ), which are the difference between the observed chemical shift and the predicted chemical shift for a given residue in a random coil. The random coil chemical shifts were generated using the neighbor-corrected IDP library (Tamiola et al. 2010), which takes into account the influence on the chemical shift of amino acid *i* due to the chemical composition of the *i* ± 1 and *i* ± 2 amino acids. We observed relatively large Δδ values for most nuclei in G193, the residue immediately preceding P194, which we can attribute to its adjacency to a proline residue (Shen and Bax 2010). For most residues in both the *cis*- and *trans*-P194 states, however, the Δδ values are near zero, indicating that both states remain dynamically disordered (Fig. 3a–d).

Δδ values report on secondary structure, with significant deviations from zero over multiple residues reflecting β-strand or α-helical conformations, depending on the nucleus and the sign of Δδ. Of those available, the Cα and Cβ shifts are the most sensitive to residual secondary structure. Both the *cis*- and *trans*-P194 ensembles show a small tendency for increased and decreased Cα and Cβ chemical shifts, respectively, in the region 180–185, which indicates a low population of β-strands in both ensembles. For residues 193–199 in the vicinity of the conserved IXI/V motif, *cis*-P194 induced a small population of β-strand, whereas the *trans*-P194 ensemble is more disordered in this region. These findings were supported by calculation of the secondary structure propensity (SSP) (Marsh et al. 2006), where the propensity for β-strand formation was estimated to be on the order of 10% for residues 195–198 in the *cis*-P194 state (Fig. 3e). Similarly, using the software δ2D (Camilloni et al. 2012), we calculated an increased population of β-strand conformations in the region near *cis*-P194, albeit with a significantly higher propensity for β-strand formation than predicted by SSP (Fig. S3, compare to Fig. S4). The increase in β-strand conformation is accompanied by a concomitant loss of polyproline-II structure in the *cis* conformation. Polyproline-II conformations populate similar angles in the Ramachandran map as β-strands, but form a distinct hydrogen bonding pattern. Thus, since the CTR interacts with the ACD in a β-strand conformation, the formation of residual β-strand structure in the CTR would be expected to influence binding to the hydrophobic β4-β8 groove (Laganowsky et al. 2010; Laganowsky and Eisenberg 2010; Hochberg et al. 2014).

### Both proline isomers are highly dynamic

**Fig. 4:**
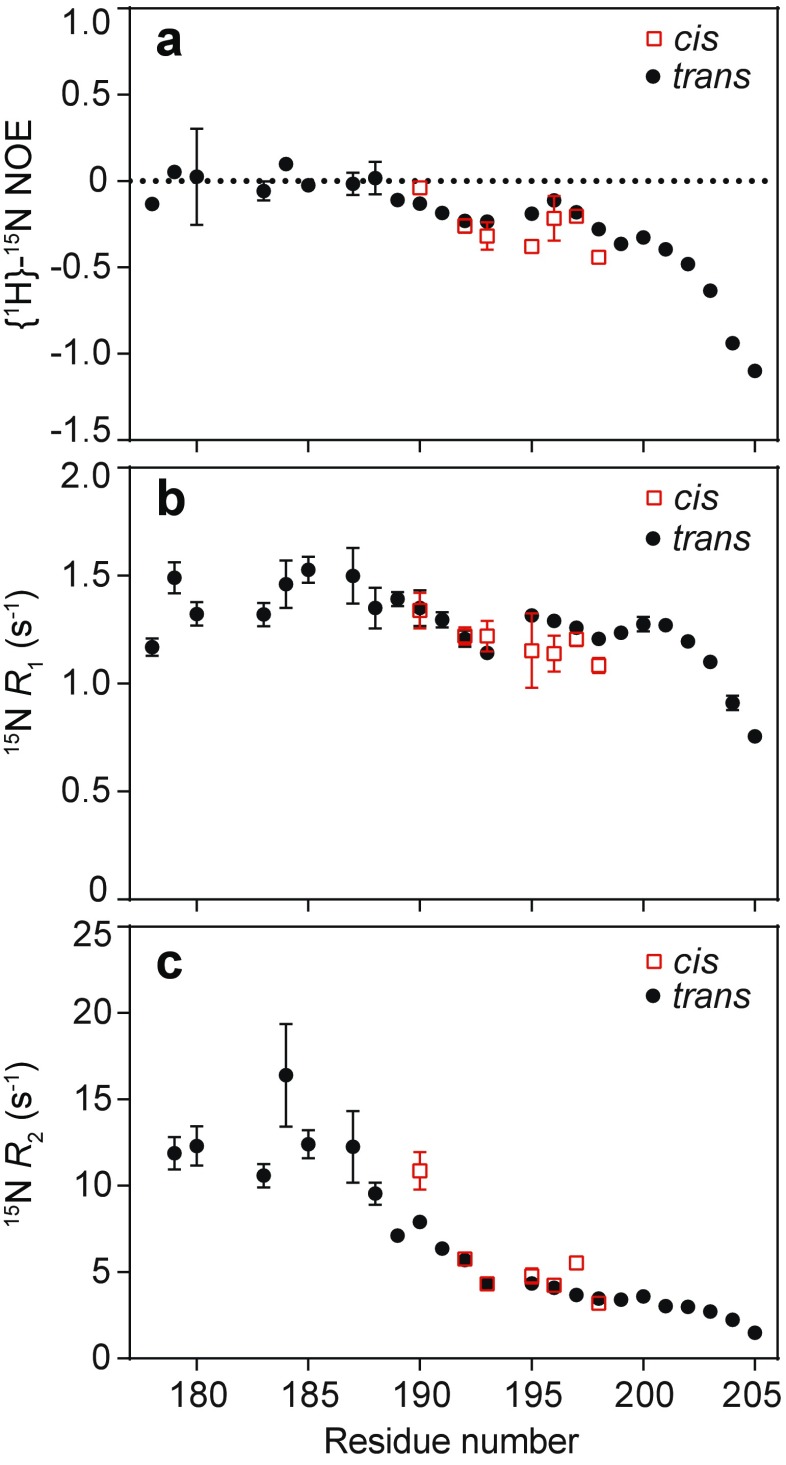
Comparison of backbone motions for the *cis*- and *trans*-Pro conformations in the C-terminal region of HSP27. **a**–**c** ^15^N spin relaxation data obtained for both *cis*- (*red*) and *trans*-P194 (*black*) HSP27. In panels **a**, the {^1^H}-^15^N nuclear Overhauser enhancement (hetNOE), **b** ^15^N longitudinal relaxation rate (*R*_1_), and **c** the ^15^N transverse relaxation rate are shown. All data were recorded at 600 MHz and 298 K

To further characterize the potential impact of *cis*-Pro formation on the internal dynamics of the CTR, we acquired ^15^N spin relaxation data that probe motions on the pico-nanosecond timescale (Jarymowycz and Stone 2006). These rapid motions reflect fluctuations of N–H bond vectors over time, providing insight into the conformational entropy and local dynamics of the involved residues. For each residue in the CTR, we measured the {^1^H}-^15^N nuclear Overhauser enhancement (hetNOE) value, and longitudinal (*R*_1_) and transverse (*R*_2_) ^15^N relaxation rates (Fig. 4a–c). While these data collectively provide a description of the fast dynamics associated with each N–H bond vector, the hetNOE is most sensitive to picosecond dynamics (Kay et al. 1989). For folded proteins, hetNOE values typically range from 0.65 to 0.85, while relatively flexible loops and regions with enhanced picosecond dynamics exhibit lower values. Consistent with this, the hetNOE measures we obtained for the *trans*-P194 state of the CTR at 600 MHz are near zero for residues 178–188, and decrease toward a minimum of −1.2 at the C-terminus (Fig. 4a). This enhanced flexibility with increased distance from the ACD is similar to observations made in αA- and αB-crystallin (Treweek et al. 2010). The *cis*-P194 hetNOE values are very similar to those for the *trans*-P194 state, except for residues E195 and K198, which are significantly lower. The overall resemblance of hetNOE values for both proline isomer states demonstrates similar dynamical behavior, with picosecond-timescale motions throughout the CTR that increase toward the C-terminus for both conformations.

Both ^15^N *R*_1_ and ^15^N *R*_2_ are less sensitive to rapid internal dynamics than the hetNOE, yet provide information regarding motions on the nanosecond timescale that are faster than the rotational correlation time. The values obtained for the *cis*- and *trans*-P194 forms are very similar (Fig. 4b, c), with the average values of *R*_1_ and *R*_2_ of (~1.2 and 5 s^−1^, respectively) and the low *R*_2_/*R*_1_ ratio (~4, Fig. S5) indicating that both *cis-* and *trans-*P194 conformational ensembles are also highly dynamic in the nanosecond regime (Fig. 4, S4, S5). In addition to these motions in the CTR of HSP27, it is possible, as in the case of αB-crystallin (Baldwin et al. 2011a), that there are contributions from conformational exchange to the observed *R*_2_ values in the IPV motif. Taken together, our data have revealed that the CTR, including the IPV motif, is highly dynamic on the picosecond-nanosecond timescale, with the magnitude of these motions increasing upon moving away from the ACD toward the C-terminus.

## Discussion

### Dynamic fluctuations of the CTR

Here we have shown that the final 28 residues in the CTR of the human sHSP HSP27 can be readily discerned by NMR spectroscopy using non-deuterated samples. The observation of their chemical shifts in the context of the 100–700-kDa oligomeric ensemble, combined with detailed analysis of chemical shifts and relaxation properties, reveals this region of HSP27 to be highly dynamic. These conclusions are in agreement with previous NMR studies of the flexible CTRs of mammalian sHSPs αA- and αB-crystallin (Carver et al. 1992; 1995b; Smulders et al. 1996; Carver and Lindner 1998; Treweek et al. 2010; Baldwin et al. 2012; Mainz et al. 2015; Delbecq et al. 2015), HSP27 (Carver and Lindner 1998), Hsp20 (van de Klundert et al. 1998), and Hsp25 (Carver et al. 1995a; Lindner et al. 2000) in which the final 10–15 residues were amenable to NMR investigation under non-heat-shock conditions.

In this study, we were able to observe and assign an additional 10 residues within the disordered CTR of HSP27 over those described previously (Carver and Lindner 1998), likely due to our usage of modern hardware, a higher magnetic field strength, and [*U*-^13^C,^15^N]-labeled samples, and we could thus probe the conformations and dynamics of the final 28 residues. Similarly, a recent NMR investigation of the CTR in human αB-crystallin was able to extend the number of observed residues to ~25 (Mainz et al. 2015). The extensions of these two sHSPs are therefore both predominantly disordered at physiological solution conditions. Notably, the IPI/V motifs in both cases are primarily detached from the oligomers, and therefore only make transient interactions with their own ACD or adjacent ACDs. Studies on αΑ- and αB-crystallin CTRs have demonstrated that rapid motions take place on the pico-nanosecond timescale (Treweek et al. 2010), and quantitative analysis of the relaxation properties of HSP25 Pro-194 reveals that its CTR has high motional flexibility (Esposito et al. 1998). We have found by ^15^N-relaxation that the disordered CTR of HSP27 also exhibits similar motions on the same timescale. These combined observations show that the motions at the end of several mammalian CTRs are effectively uncoupled from the slowly tumbling oligomers, such that they behave comparably to an unstructured peptide.

### *Cis*-*trans* proline isomerization in the CTR

We identified that slow inter-conversion between *cis*- and *trans*-proline conformations within the G193-P194 peptide bond takes place in HSP27. Proline isomerization has also been reported in the extension of bovine αB-crystallin, where the authors were able to attribute the two peaks for V169 to *cis* and *trans* conformers of the K166-P167 peptide bond (Carver and Lindner 1998). The second proline residue in the CTR of HSP27 is situated within the highly conserved IPV motif. We cannot conclude for certain that *cis*-*trans* isomerization of P182 takes place since the chemical shifts near this residue are weak, rendering a lowly populated *cis* conformation unobservable. However, using methyl-based experiments on a peptide encompassing the IPV motif, we observed more NMR signals than would be expected from the amino acid sequence, which likely indicates *cis*-*trans* isomerization about the I181-P182 peptide bond. In support of this, we note that, when taking advantage of the enhanced signal-to-noise afforded by the use of perdeuterated protein, additional weak resonances were detected for residues near P160 in the IPI motif of human αB-crystallin (Mainz et al. 2015). This combined evidence suggests that both HSP27 and αB-crystallin undergo *cis*-*trans* proline isomerization in the IPI/V motif.

**Fig. 5:**
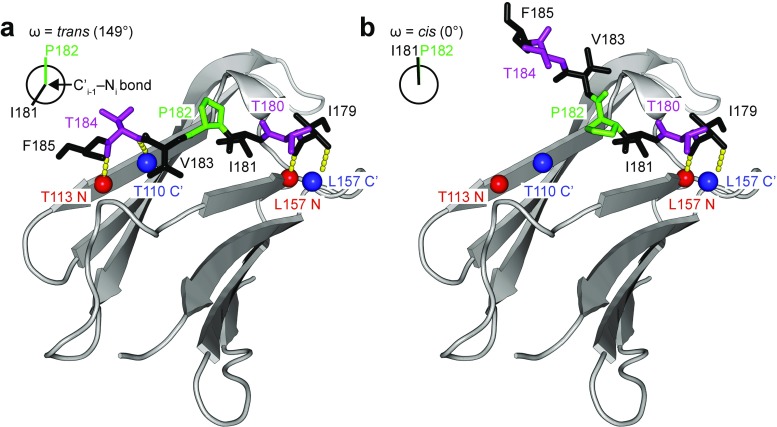
Impact of *cis*-*trans* proline isomerization on CTR binding to HSP27. **a** Crystal structure of the HSP27 C-terminal peptide (I179-F185; PDB 4MJH) bound to its ACD. The CTR peptide is colored according to the scheme in Fig. 1. Hydrophobic interactions (I181, V183) and hydrogen bonds (*yellow dashes*) between the CTR and ACD stabilize binding. The central I181-P182 peptide bond is in the *trans*-conformation, as indicated by the schematic of the dihedral angle ω in the *top left corner*. When viewed down the axis of the peptide bond, the angle between the C′-Cα bond from I181 and the N-Cα bond from P182 is 149°, similar to the value expected for a *trans* peptide bond (ω = 180°). **b** If the I181-P182 peptide bond was rotated into the *cis* conformation (ω = 0°), binding of the CTR would be energetically less favorable. In the *cis* conformation, two hydrogen bonds involving the CTR and the β4-strand would be broken and the hydrophobic side-chains of V183 and F185 would be exposed to solvent

In HSP27, we found that the *cis*-P194 conformation is populated to ~15% at near-physiological solution conditions. Although both *cis*- and *trans*-P194 forms are highly dynamic, we determined that both proline states of the CTR ensembles have a theoretical tendency of ~10% to adopt β-strand conformations between residues 180 and 185, encompassing the IPV motif. It is interesting to speculate on the significance of the residual β-strand structure in this region, as crystal structures of ACD-CTR complexes reveal an extended, *trans*-conformation of the CTR. The structure of the HSP27-CTR complex shows that numerous hydrogen bonds are made between the β4 and β8 strands of the ACD and polar atoms in the CTR (Fig. 5a). Thus, formation of residual β-strand structure in residues 180–185 could facilitate both docking of the hydrophobic IPI/V motif and hydrogen bonding between the ACD and CTR. Alternatively, the residual structure could also enable inter-molecular interactions with another sHSP CTR, or a substrate protein in a similar conformation. In addition, we note that *cis*-P194 leads to a similar increase in β-strand propensity between residues 193 and 199. The residual structure within the *cis*-P194 state could have functional implications in regulating oligomerization or substrate interactions. This hypothesis is supported by the presence of mutations near P194 that lead to Charcot-Marie-Tooth disease (R188W) (Capponi et al. 2011) and amyotrophic lateral sclerosis (Q190H) (Capponi et al. 2016).

*Cis*-*trans* proline isomerization about the I181-P182 bond could play a significant role in HSP27 oligomerization. The conformation of the CTR when bound in the hydrophobic groove of the ACD is expected to be in the extended *trans*-P182 form, as has been observed in structures of other sHSP oligomers (Hilton et al. 2013b) and for the CTR peptide co-crystallized with the HSP27 ACD (Fig. 5a) (Hochberg et al. 2014). In HSP27, the I181-P182 peptide bond in the CTR adopts a *trans*-conformation, which allows the side-chains of I181 and V183 to dock into the hydrophobic groove created by the β4 and β8 strands (Fig. 5a). Rotation about the I181-P182 peptide bond to create a *cis* conformation would create a less energetically favorable state in which the hydrogen bonds with the β4 strand are broken and V183 and F185 are exposed to solvent (Fig. 5b). Therefore, we would expect that the *trans*-P182 form has a higher affinity for the ACD than the *cis* form. For the *trans*-I181-P182 state that contains residual β-strand conformation (~10%), this minor population could comprise a binding-compatible state. Additionally, any PTMs and mutations that affect the ratio and inter-conversion rate of these two forms could potentially affect interactions with the ACD. Notably, three inheritable mutations (T180I, P182S, and P182L) in this region lead to the onset of the motor neuropathy Charcot-Marie-Tooth disease (D’Ydewalle et al. 2011; Chalova et al. 2014).

The CTR of αB-crystallin also has a central proline residue within its IXI/V motif that could be prone to *cis*-*trans* isomerization. We previously found that this motif was also predominately disordered, and quantified its kinetics and thermodynamics of binding to the ACD of αB-crystallin using a combination of methyl-TROSY CPMG NMR experiments and native MS (Baldwin et al. 2011a, b). The lifetime of the bound ACD-CTR αB-crystallin complex varied strongly with temperature and pH, but was typically on the order of milliseconds at near-physiological conditions, and populated to approximately 1% (Baldwin et al. 2011a). However, the exchange of subunits between oligomers is mediated by monomers with lifetimes on the order of seconds under similar conditions (Baldwin et al. 2011b), much longer than CTR binding. Despite the differences in lifetime of 3 orders of magnitude, CTR binding and subunit exchange are kinetically coupled (Baldwin et al. 2011a), with the independent but concomitant binding of two adjacent CTRs occurring on the seconds timescale, and being necessary for the dissociation of a monomer from an oligomer (Baldwin et al. 2012; Hochberg and Benesch 2014).

The rate for *cis-trans* proline isomerization would be expected to be an additional 3 orders of magnitude slower, with lifetimes on the tens of minutes timescale (Wedemeyer et al. 2002). As the *cis-trans* isomerization rates are 6 orders of magnitude slower than the CTR binding process, it is unlikely that these events are directly correlated. It is interesting, however, to speculate that the two different proline conformers may have different affinities for the ACD. The *trans* form would perhaps be expected to bind the ACD transiently a large number of times during its lifetime before conversion to the relatively inert *cis* conformation. Consistent with this, mutation of the central proline has been shown to modulate both the kinetics and thermodynamics of oligomerization of αB-crystallin, resulting in slightly faster subunit exchange rates and an altered equilibrium oligomeric distribution (Hilton et al. 2013a). These results reveal that, while the conserved IXI/V motif within the CTR is predominantly disordered, it can nevertheless exert significant regulatory properties on the underlying oligomers by affecting the transiently populated bound states. As such, effects such as *cis-trans* proline isomerization may, in principle, play an important regulatory role.

### Possible roles for *cis*-*trans* proline isomerization

Inside cells, *cis*-proline bonds play fundamental roles with widespread biological implications (Sarkar et al. 2007). The slow spontaneous rate of *cis*-*trans* proline isomerization often regulates the rates with which proteins fold (Wedemeyer et al. 2002) and *cis*-proline bonds can induce structural changes in membrane-embedded receptors (Lummis et al. 2005). A specific class of ubiquitous molecular chaperones, peptidyl-prolyl isomerases, such as trigger factor, function to catalyze the otherwise slow inherent inter-conversion of *cis*- and *trans*-Pro states (Gothel and Marahiel 1999). In HSP70, it has been proposed that a *cis*-Pro bond involving a universally conserved proline residue in the ATPase domain regulates the allosteric transition between nucleotide- and substrate-bound states (Vogel et al. 2006; Alderson et al. 2016). Considering the evidence of allosteric communication between the contact the CTR transiently makes with the ACD, and the dimer interface (Delbecq et al. 2012; Hochberg and Benesch 2014), we speculate that the *cis*-P182 and *cis*-P194 bonds could affect the oligomerization of HSP27, or even its interactions with cellular targets. In addition, the post-translational modification of residues in the CTR, such as the non-enzymatic argipyrimidation or R188 (Sakamoto et al. 2002), could be influenced by isomerization regulating solvent exposure.

### Conclusions

We have employed solution-state NMR spectroscopy to probe the conformation and dynamics of the disordered CTR of HSP27. We found that the CTR contains residual β-strand structure in the highly conserved IPV motif, which could impact its subsequent binding to the ACD. In addition, our results demonstrate that both proline residues (P182, P194) within the CTR undergo *cis*-*trans* isomerization, with the *cis*-P194 conformation retaining its rapid internal dynamics, but leading to an increase in residual β-strand structure in residues 193–199. We speculate that *cis*-*trans* proline isomerization is a feature of those mammalian sHSP CTRs that are flexible, in particular HSP27 and αB-crystallin. Given that these two are the human sHSPs with the most widespread expression, are heavily up-regulated during stress, and have the largest number of interactors (Kampinga et al. 2015; Mymrikov et al. 2017), it is likely that the dynamic fluctuations of their CTRs play an important role in their canonical sHSP chaperone activity.

## Materials and methods

### HSP27 expression and purification from *Escherichia coli*

An ampicillin-resistant (Amp^R^) HSP27 plasmid was kindly donated by Prof. Heath Ecroyd (University of Wollongong). The HSP27 plasmid was transformed into BL21-Gold(DE3) competent cells and plated on an LB-agar plate containing 100 μg mL^−1^ of Amp. A single colony from the LB-agar plate was used to inoculate a 5-mL culture of LB medium with 100 μg mL^−1^ of Amp for expression of full-length HSP27. Following growth at 37 °C for 6 h, the 5-mL culture was transferred to 100 mL (LB medium) or 50 mL (M9 minimal medium) cultures and grown overnight at 37 °C, which were then used to inoculate 1 L (LB medium) or 500 mL (M9 minimal medium) cultures. The M9 minimal medium consisted of 6 g L^−1^ Na_2_HPO_4_, 3 g L^−1^ KH_2_PO_4_, 1 g L^−1^ NaCl, 1 g L^−1 15^NH_4_Cl, 6 g L^−1^ natural abundance glucose, 1 mM MgSO_4_, and 100 μΜ CaCl_2_. For preparation of uniformly-^13^C, ^15^N-labeled ([*U*-^13^C,^15^N]-) HSP27, the M9 minimal medium contained 2 g L^−1^ of [*U*-^13^C]-glucose and 1 g L^−1^ of ^15^NH_4_Cl. When the absorbance at 600 nm of these cultures reached between 0.6 and 0.8 units, IPTG was added to a final concentration of 100 μg mL^−1^ and protein expression ensued for 3 h at 37 °C. Cells were pelleted and frozen at −80 °C until use.

HSP27 was purified in a similar manner as described previously (Muranova et al. 2015) using anion exchange chromatography (AEX) with HiTrapQ HP columns (GE Healthcare) followed by size exclusion chromatography (SEC) on a Superdex S200 column (GE Healthcare). The final SEC buffer contained 20 mM Tris-HCl, 150 mM NaCl, 2 mM EDTA at pH 7.0. After pooling HSP27-containing fractions that eluted from the Superdex S200 column, the samples were further purified with a second AEX step involving the usage of a HiTrap Capto Q ImpRes column (GE Healthcare). HSP27 was then buffer-exchanged using Amicon spin filters (Millipore) into 30 mM NaH_2_PO_4_, 100 mM NaCl, 2 mM EDTA, 2 mM NaN_3_, pH 7.0 with 6% D_2_O (NMR buffer with 100 mM NaCl).

### Native mass spectrometry of HSP27

A 25-μM sample of natural abundance HSP27 was buffer exchanged into 200 mM ammonium acetate, pH 6.9. Data were collected on an ion-mobility Synapt G1 mass spectrometer (Waters) under ionization conditions that maintain non-covalent interactions, according to a protocol described previously (Kondrat et al. 2015).

### Backbone NMR assignments for [*U*-^13^C,^15^N]-HSP27

All NMR spectroscopy experiments for resonance assignments of HSP27 were recorded on a 600-MHz Varian-Inova spectrometer equipped with a 5-mm z-axis gradient triple resonance, room temperature probe. [*U*-^13^C,^15^N]-HSP27 was prepared at a final concentration of 2 mM in NMR buffer with 100 mM NaCl. Standard assignment experiments were collected, namely 3D HNCO, HN(CO)CA, HN(CA)CO, HNCA, HNCACB, and C(CO)NH spectra (Sattler et al. 1999), and these experiments contained either uniform sampling or non-uniform sampling (NUS) schemes in the indirect dimensions. When NUS was employed, data were recorded with ~25% sparsity in the indirect dimensions and time-domain data were reconstructed with MddNMR (Kazimierczuk and Orekhov 2011). All 3D NMR spectra were acquired at 25 °C, processed with NMRPipe (Delaglio et al. 1995), and visualized with Sparky (Lee et al. 2015).

### Natural abundance ^13^C NMR spectra of the HSP27 C-terminal tail peptide

A peptide encompassing the IPI/V motif of HSP27 was ordered from BioMatik. The sequence of the peptide was EITIPVTFE, with an N^α^ acetyl group at the N-terminus. Approximately 5 mg of peptide was dissolved in 600 μL of 30 mM NaH_2_PO_4_ at pH 12 to ensure solubility of the hydrophobic peptide. pH values below 12 led to precipitation of the peptide at the high concentrations that were required to detect ^13^C at natural abundance. A 2D ^1^H-^13^C HSQC spectrum was recorded on a 500-MHz Bruker Avance III spectrometer equipped with a cryogenically cooled probe. Since ^13^C is present at natural abundance to only 1% ^13^C, a minor *cis* population (~10%) would yield an effective NMR-observable concentration of only 0.1% of the total peptide. Thus from the 5 mg that were dissolved in NMR buffer at a concentration of ~8 mM, the effective total concentration of NMR-observable peptide is 80 μM and with an effective concentration of signals from a minor *cis*-P182 state near 8 μM.

### Analysis of residual secondary structure in the HSP27 C-terminal region

To analyze residual secondary structure in the CTR of HSP27, we utilized the software programs SSP (Marsh et al. 2006) and δ2D (Camilloni et al. 2012). For both SSP and δ2D, calculations were performed with ^13^Cα, ^13^Cβ, and ^13^CO chemical shifts or with ^13^Cα, ^13^Cβ, ^13^CO, ^15^N, and ^1^H^N^ chemical shifts for both the *cis*-P194 and *trans*-P194 conformations.

### ^15^N spin relaxation experiments (R_1_, R_2_, NOE) on HSP27

Standard pulse sequences to measure hetNOE, longitudinal (*T*_1_), and transverse (*T*_2_) relaxation times were employed (Palmer 1993). *T*_1_ and *T*_2_ values are reported as their respective inverses, i.e., rates (*R*_1_ and *R*_2_), for convenience. The spectrometer temperature was calibrated with d_4_-methanol. We recorded ^15^N *R*_1_, ^15^N *R*_2_, and hetNOE datasets at 25 °C on a 2-mM sample of [*U*-^13^C,^15^N]-HSP27 in NMR buffer with 100 mM NaCl. All data sets mentioned here were processed with NMRPipe (Delaglio et al. 1995), visualized with Sparky (Lee et al. 2015), and further analyzed with FuDA (http://www.biochem.ucl.ac.uk/hansen/fuda/) to fit peak shapes. To obtain the ^15^N *R*_2_ estimated values in Fig. S6, we used the average ^15^N *R*_2_ value that was measured for a 20-kDa protein at a similar concentration (1 mM) on our spectrometer to then extrapolate to proteins that are 40 and 60 kDa in molecular mass. The ^15^N *R*_2_ is proportional to the rotational correlation time of a molecule, which is itself roughly proportional to the molecular mass, assuming isotropic rotational diffusion.

### Thermodynamic analysis of *cis-trans* G193-P194 isomerization

^1^H-^15^N HSQC spectra on [*U*-^15^ N]-HSP27 were recorded at 20, 30, and 40 °C. FuDA was used to fit peakshapes with uniform linewidths to extract reliable peak intensities. The equilibrium constant *K* was calculated as the population of *cis*-P194 divided by the population of *trans*-P194. The resultant values of *K* were fit to the van’t Hoff equation in order to extract the change in enthalpy (Δ*H*) and the change in entropy (Δ*S*) that accompany the *trans*- to *cis*-P194 reaction.

## Electronic supplementary material

ESM 1 (DOC 1011 kb)
