# Supplementary material for: Proline isomerization in the C-terminal region of HSP27
Source: Cell Stress Chaperones. 2017 May 25;22(4):639–51. doi: 10.1007/s12192-017-0791-z (PMC5465039; doi:10.1007/s12192-017-0791-z)
Supplement: Supplementary file 1 — (DOC 1011 kb) [file 12192_2017_791_MOESM1_ESM.doc]

**Supporting information for:**

**Proline isomerization in the**

**C-terminal region of HSP27**

T. Reid Alderson^1,2^, Justin L. P. Benesch^1†^, Andrew J. Baldwin^1†^

*^1^Department of Chemistry, Physical and Theoretical Chemistry Laboratory, University of Oxford, South Parks Road, Oxford, OX1 3QZ, U. K.*

*^2^Laboratory of Chemical Physics, National Institutes of Diabetes and Digestive and Kidney Diseases, National Institutes of Health, Bethesda, MD, 20892, U. S. A.*

^†^Correspondence to: justin.benesch@chem.ox.ac.uk, +44 1865 285420;

andrew.baldwin@chem.ox.ac.uk , +44 1865 275420

**Supporting Figures**


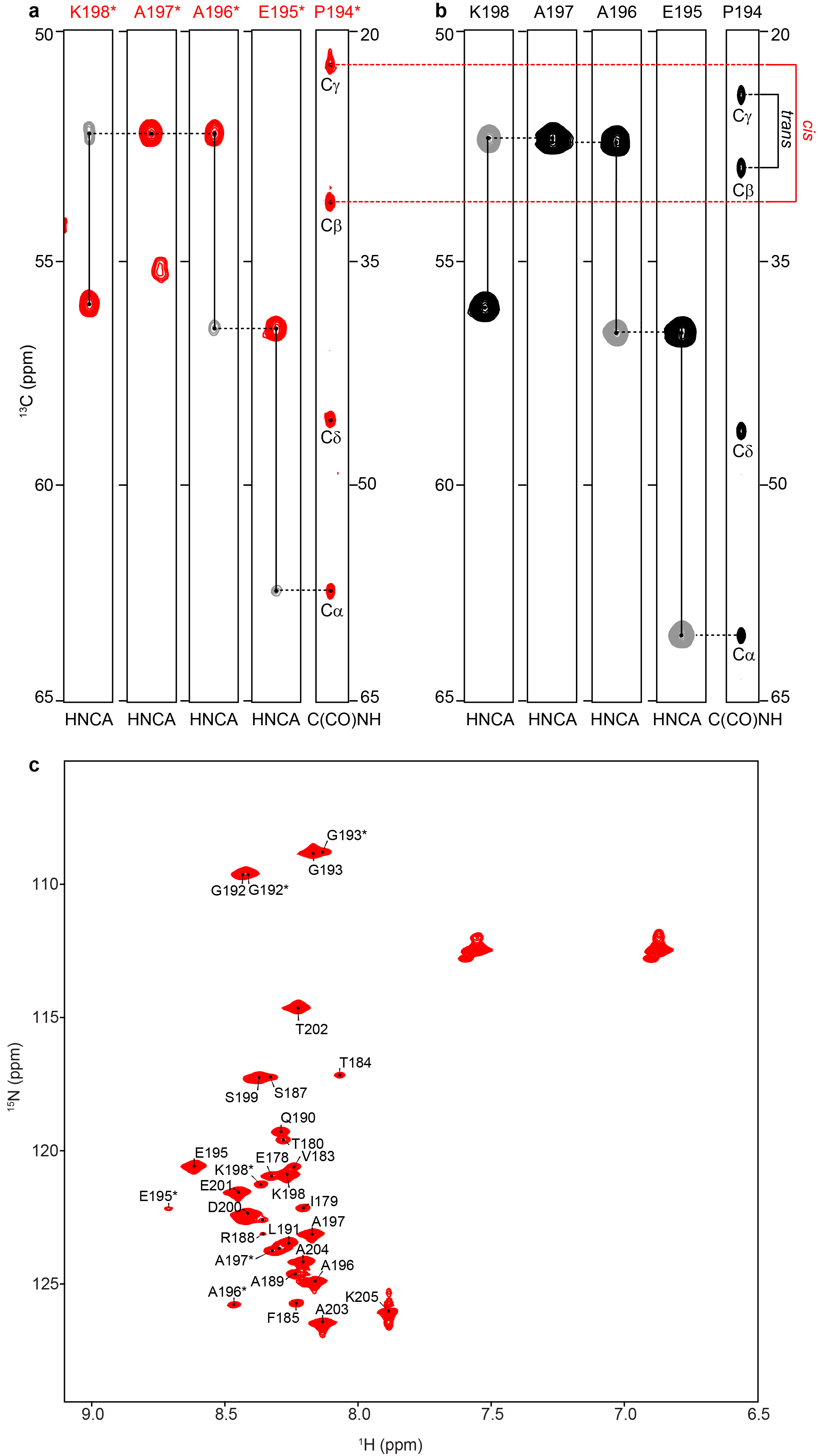


**Figure S1 2D strip plots from 3D NMR spectra that were used to assign the *cis*- and *trans*-Pro conformations of the HSP27 C-terminal region.** 2D strip plots from 3D HNCA and 3D C(CO)NH spectra from [*U*-^13^C,^15^N]-HSP27. The 2D strips were ­taken at the ^15^N frequency of the residue listed above the HNCA strip. For the C(CO)NH strips, the ^15^N frequency is at E195. (**a**) Strip plots from *cis*-P194 showing the sequential assignment of residues that exist in the *cis*-P194 conformation. Grey peaks indicate resonances from the preceding amino acid and red peaks indicate resonances from the present amino acid. The C(CO)NH strip indicates the ^13^C side-chain chemical shifts of P194. (**b**) Depicts the same strips as in (**a**), except that these resonances arise from *trans*-P194. Note the difference between the ^13^Cβ and ^13^Cδ chemical shifts for P194 in the *cis* and *trans* states, as this difference was used to assign P194 to either *cis* (10 ppm) or trans (5 ppm). (**c**) ^1^H-^15^N HSQC spectrum of HSP27 plotted at a lower contour level, such that the peak arising from E195 in the *cis*-P194 state is now visible. Doubled peaks that are indicated with an asterisk (*) and unassigned peaks have no label associated to them.

**
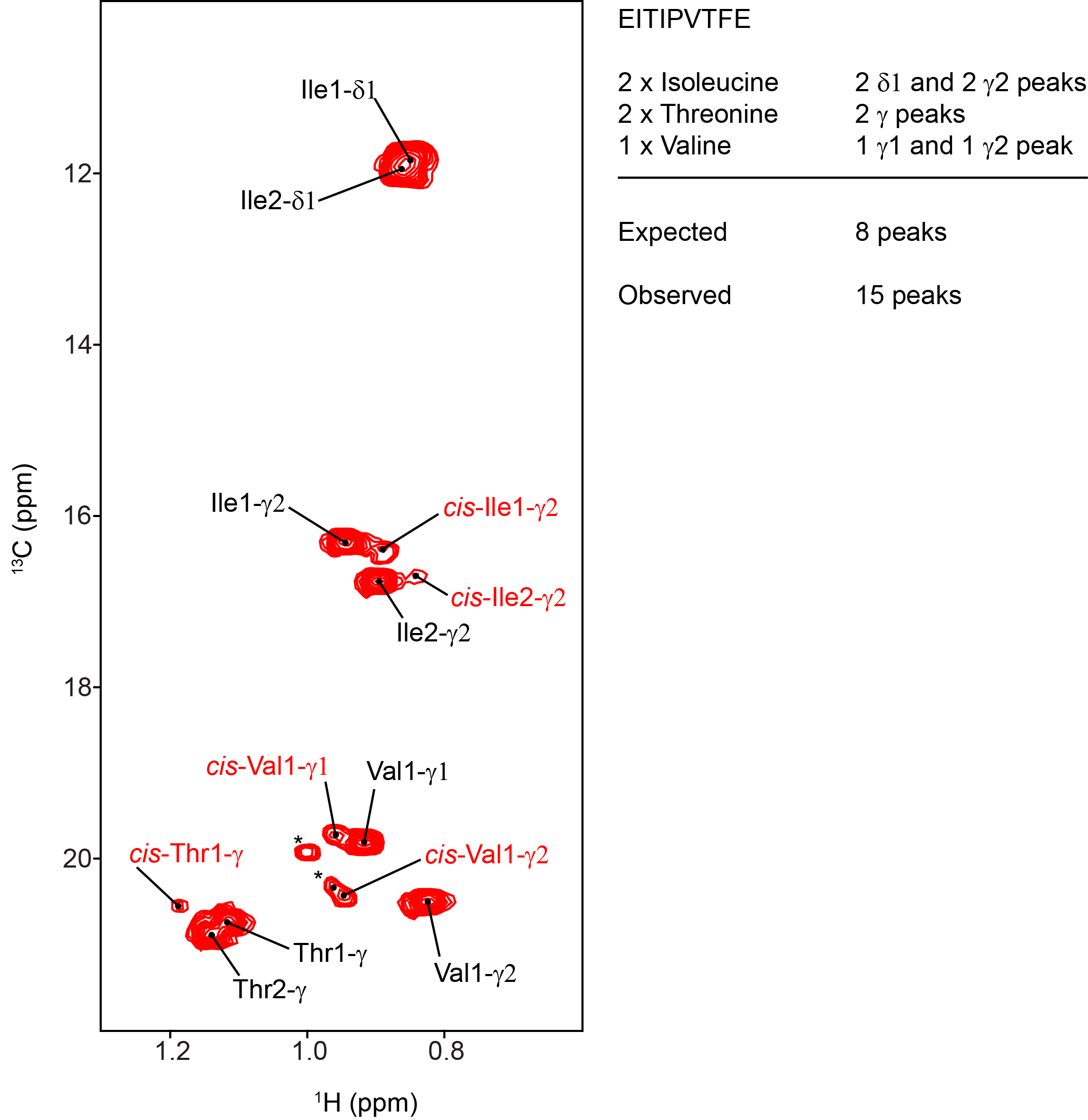
**

**Figure S2 Evidence for *cis*-*trans* proline isomerization in the IPV motif of HSP27.** A peptide encompassing the IPV motif of HSP27 (Ac-EITIPVTFE) was dissolved in NMR buffer at pH 12 to ensure solubility. Shown here is the methyl region from a ^1^H-^13^C HSQC spectrum in which eight peaks are expected on the basis of the amino acid sequence of the peptide. However, a total of 15 peaks are observed with clear doubling of resonances from both isoleucine γ2 and valine γ1 peaks, and one of the threonine γ peaks. This indicates that there are multiple conformations of the peptide present in solution with exchange between conformation that is slow on the NMR timescale (>~100 ms), similar to the behaviour of the C-terminal extension of HSP27 and the *cis*-P194 state.

**
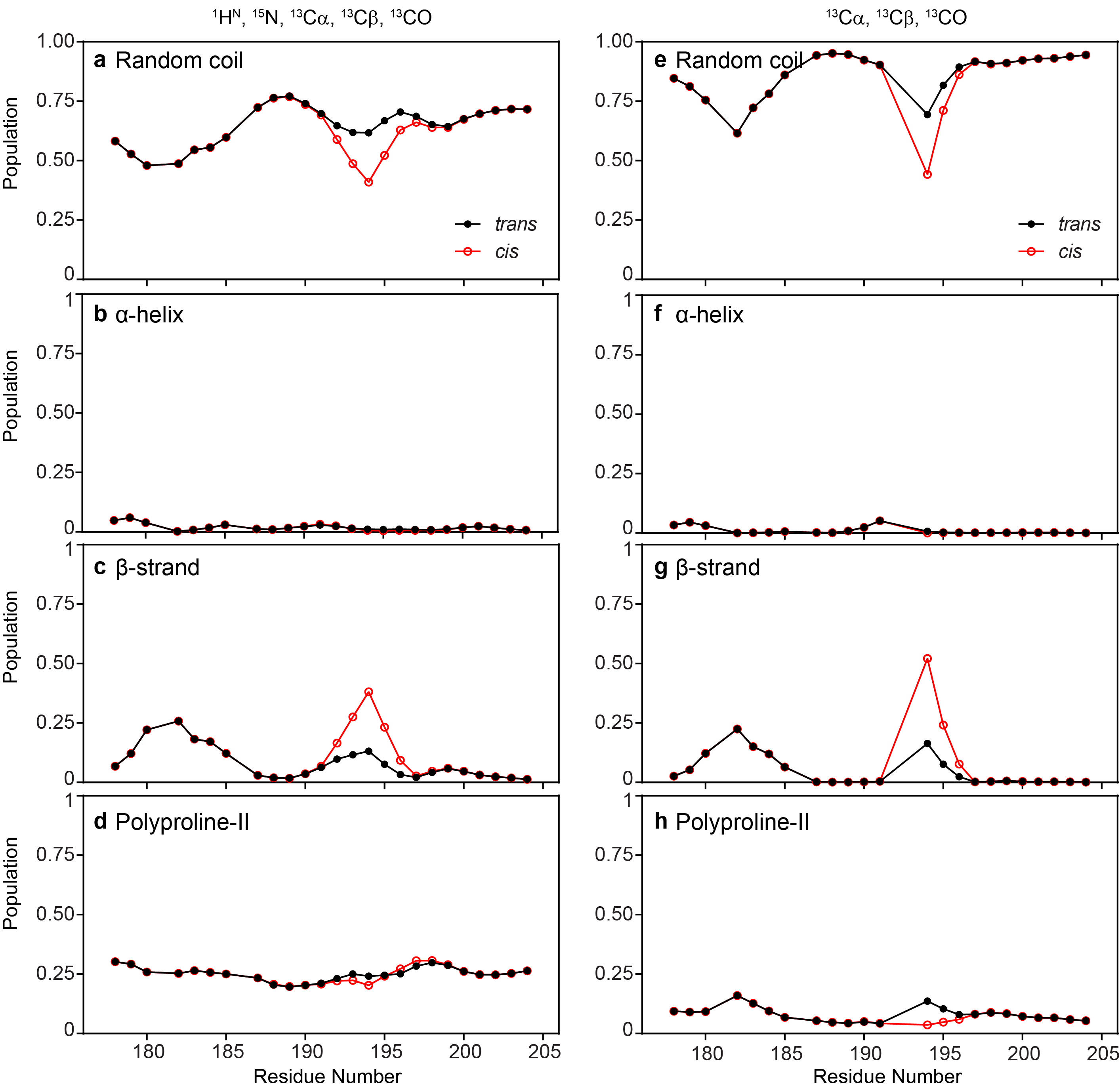
**

**Figure S3 Residual structure in the CTR of HSP27 probed by δ2D.** The assigned chemical shifts from the CTR of HSP27 were used as input for δ2D, which calculates the population of random coil (**a,e**), α-helix (**b,f**), β-strand (**c,g**), and polyproline-II (**d,h**). In panels **a-d**, all assigned chemical shifts were included in the calculation. In panels **e-h**, only ^13^C chemical shifts were used. Note that three chemical shifts are required to calculate residual secondary structure, so all glycine residues (G193, G194), which lack Cβ shifts, do not have calculated values under these conditions.

**
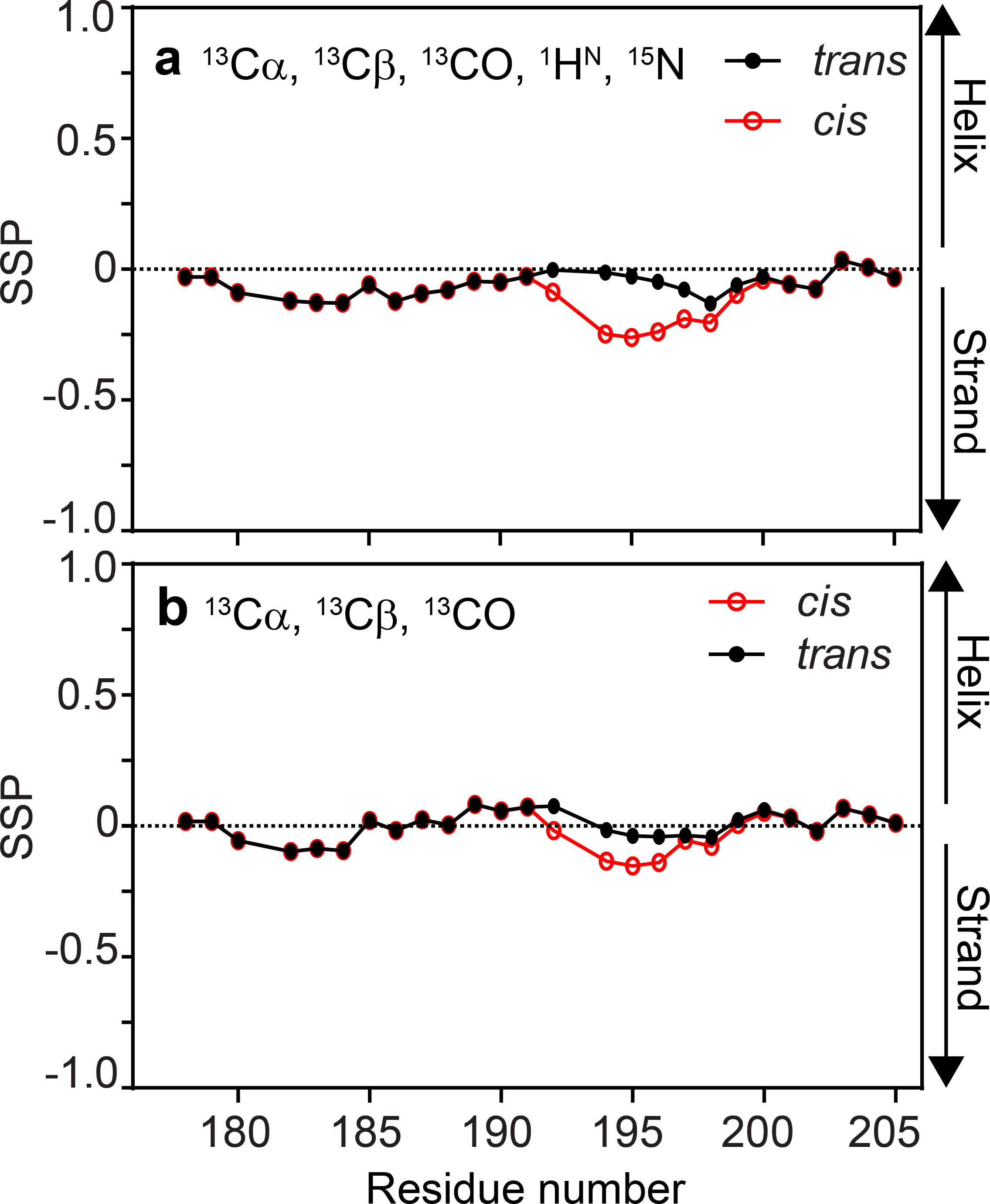
**

**Figure S4 Residual structure in the CTR of HSP27 probed by SSP**. The assigned chemical shifts from the CTR of HSP27 were used as input for SSP. (**a**) The proportion of α-helix (positive values) and β-strand (negative values) is shown when chemical shifts from all nuclei are included in the calculation. (**b**) The proportion of α-helix and β-strand when only ^13^C chemical shifts are used. Note that the proportion of β-strand decreases when only ^13^C chemical shifts are included.

**
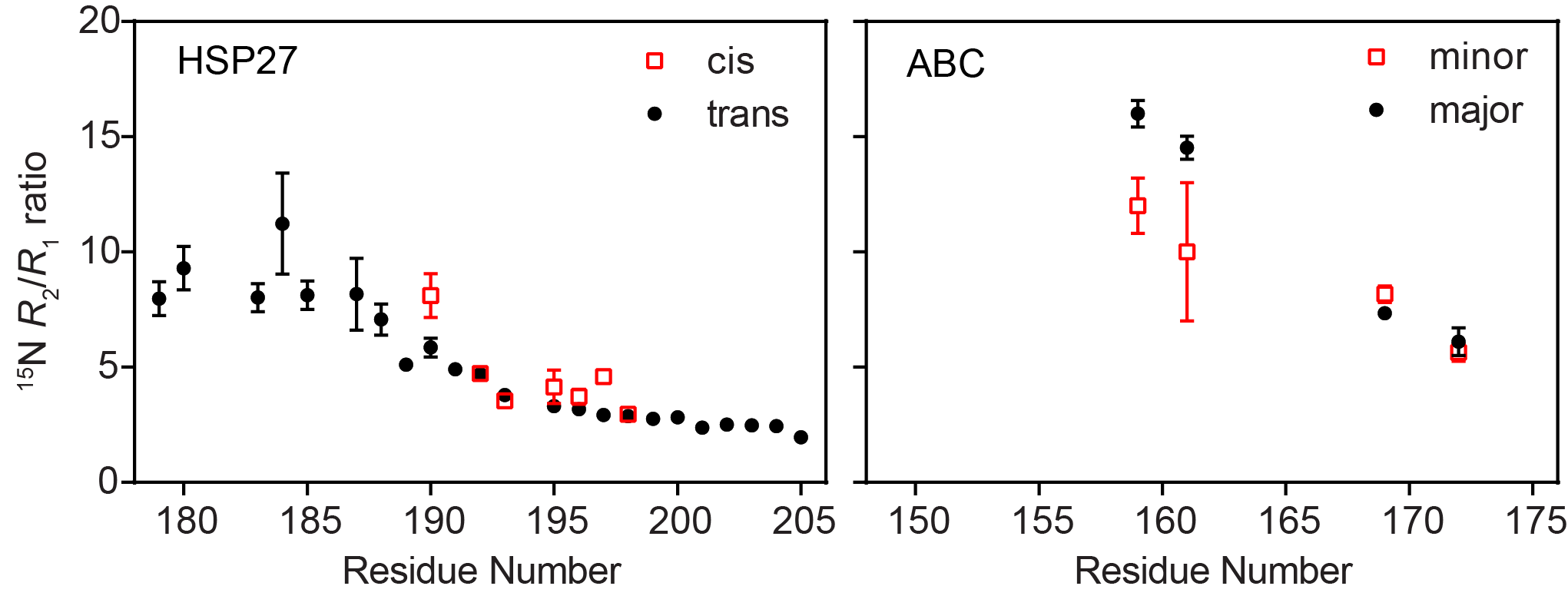
**

**Figure S5 ^15^N *R*_2_/*R*_1_ ratios for HSP27**. The ratio of ^15^N *R*_2_/*R*_1_ relaxation rates is sensitive to internal motions, with lower values signifying faster tumbling. ^15^N *R*_2_/*R*_1_ ratios are shown as a function of residue number for HSP27. In black are resonances from the *trans*-P194 state of HSP27s, and in red are the resonances from the *cis*­-P194 state of HSP27.

**
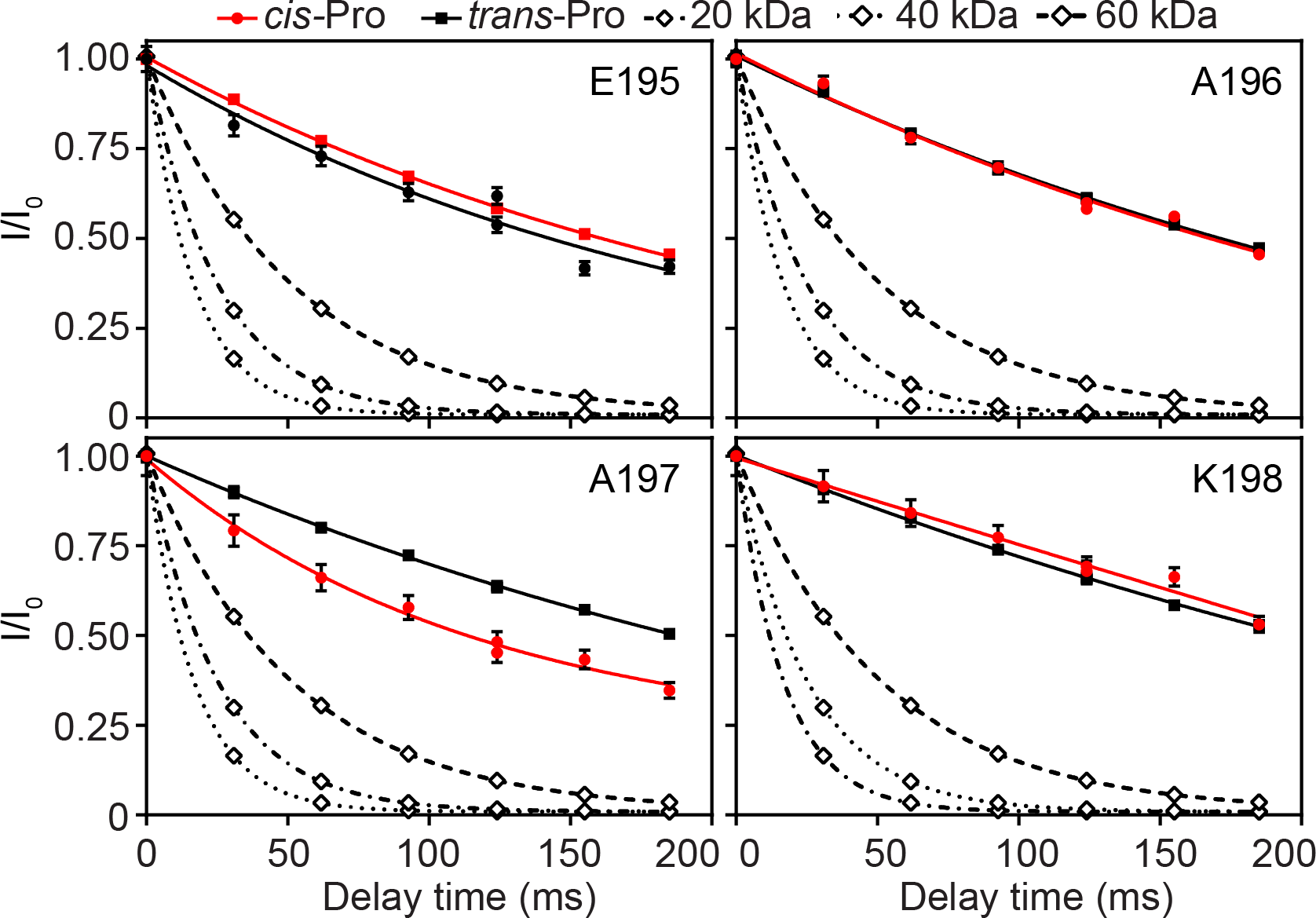
**

**Figure S6 Example transverse ^15^N relaxation data (*R*_2_) for the *cis*- and *trans*-Pro conformations of the HSP27 C-terminal domain.** Experimental (20 kDa) and simulated (40 and 60 kDa) transverse relaxation rates are also shown for comparison. Relaxation data from HSP27 are shown for resonances E195, A196, A196, and K198 for both *cis* (red) and *trans*-P194 (black) conformations. The overall similarity in ^15^N *R*_2_ values suggests that the *cis* and *trans* states exhibit similar backbone motions.
